# Low PTH Levels in Adolescents With Anorexia Nervosa

**DOI:** 10.3389/fped.2020.00099

**Published:** 2020-03-11

**Authors:** Nina Lenherr-Taube, Karin Trajcevski, Etienne Sochett, Debra K. Katzman

**Affiliations:** ^1^Division of Endocrinology, Department of Paediatrics, Hospital for Sick Children, Toronto, ON, Canada; ^2^Department of Paediatrics, University of Toronto, Toronto, ON, Canada; ^3^Division of Adolescent Medicine, Department of Paediatrics, Hospital for Sick Children, Toronto, ON, Canada

**Keywords:** PTH, bone mineral density, nephrocalcinosis, anorexia nervosa, adolescents

## Abstract

**Introduction:** Patients with anorexia nervosa (AN) experience medical complications including impaired bone metabolism, increased fracture rate, kidney stones and chronic renal failure. However, the mechanisms of such complications are not fully understood. Healthy adolescents have been shown to have higher PTH levels when compared with pre-pubertal children and adults. Given the importance of central measures of calcium and vitamin D metabolism in bone and kidney health, 25-hydroxyvitamin D (25OHD) and parathyroid hormone (PTH) have been extensively investigated in patients with AN, however none of the previous studies accounted for age-specific reference ranges for PTH. The aim of this study was to investigate central measures of calcium and vitamin D metabolism in adolescents with newly diagnosed AN using age-specific reference ranges and to determine whether any significant abnormalities required further study.

**Methods:** This was a cross-sectional study of 61 adolescents (mean age = aged 15.2 ± 1.56 years) with newly diagnosed AN, referred to a tertiary center over a period of 2 years. Demographic, auxiological, and nutrient (vitamin D and calcium) intake data was obtained. Central measures of calcium and vitamin D metabolism in blood and urine were investigated. PTH results were compared with age-specific reference ranges from the Canadian Laboratory Initiative on Pediatric Reference Intervals (CALIPER). Descriptive statistics and correlation analysis were performed.

**Results:** Low PTH levels were observed in 35% of the cohort. Overall, serum calcium, phosphate and 25OHD were within the reference range. Using loess curves, PTH had a significant negative and non-linear correlation with 25OHD with an inflection point at a 25OHD level of 100 nmol/l, above which the association was no longer present. Correlation analysis did not show a significant association between PTH and total or corrected serum calcium, urine calcium/creatinine (Ca/Cr) ratio, total dietary calcium intake, magnesium or Tanner staging.

**Conclusion:** PTH levels were reduced in approximately a third of adolescents with AN. This observation has not been reported given the universal usage of reference ranges that covers all ages. This finding may unmask a potential role for reduced PTH levels in the pathogenesis of kidney stones and bone phenotype in patients with AN.

## Introduction

Anorexia nervosa (AN) is characterized by restriction of energy intake leading to low body weight, intense fear of gaining weight or becoming fat, and disturbances in the perception of body weight and shape (1) and is associated with alterations in multiple endocrine axes. These alterations are thought to preserve energy and protect essential physiological systems (2–4).

Patients with AN are known to experience both bone and renal complications including impaired bone metabolism, increased fracture rate, kidney stones, hypercalciuria and chronic renal failure (5–9). Hormonal changes including hypothalamic hypogonadism, growth hormone resistance, reduced insulin-like growth factor-1 (IGF-1) production/release and changes in neuropeptides that mediate appetite regulation, are known to contribute to impaired bone health in adolescents with AN (2, 6, 10, 11). However, the mechanisms and associations between impaired bone health and renal complications have not been fully elucidated (5, 12–16).

Given the importance of central measures of calcium and vitamin D metabolism in bone and kidney health, blood levels of 25-hydroxyvitamin D (25OHD) and parathyroid hormone (PTH) have been widely investigated in these patients, yet studies have reported inconsistent results (2, 4, 10, 17, 18).

Levels of PTH are known to be affected by several variables, including 25OHD, calcium intake, body mass index (BMI) and age. Within the pediatric population, PTH levels are higher in pubertal compared to pre-pubertal children (19, 20). When the lower end of the reference range is examined, adolescents have a higher lower limit compared to both adults and younger children (19). These data were recently confirmed by the age-specific reference ranges from the Canadian Laboratory Initiative on Pediatric Reference Intervals (CALIPER) (21). Using the IDS-iSYS PTH assay, the lower limit of PTH in adolescents (aged 9–17 years) was 22 pg/ml (21) compared to all ages was 12 pg/ml (22).

A subset of patients with AN [17.6% (23, 24) to 88% (12)] have been found to exhibit low levels of 25OHD, despite relatively normal levels of PTH. By contrast, other observational and interventional studies have found no significant difference in PTH levels in patients with AN compared to controls (4, 10, 11, 14, 17, 25–29). One study (18) reported lower PTH levels while another study (2) reported higher PTH levels in adolescents with AN. However, to our knowledge none of these studies accounted for age-specific reference ranges for PTH. These conflicting results suggest that further studies are needed to assess central measures of calcium and vitamin D metabolites in adolescent patients with AN, particularly 25OHD and PTH.

The aim of this study was to investigate central measures of calcium and vitamin D metabolism in adolescents with newly diagnosed AN using age-specific reference ranges and to determine whether any significant abnormalities required further study.

## Methods

### Study Design

This was a cross-sectional study including all new patients between 11-and 17-years old referred to the Eating Disorder Program (EDP) at the Hospital for Sick Children (SickKids) between July 2011 and November 2013. Patients were included if they met the DSM-IV criteria for AN as determined by a standard diagnostic protocol in the EDP at SickKids (30). Patients were excluded if they had another chronic disease, were taking medications known to affect bone metabolism or which might interact with vitamin D supplements, had skin conditions that would affect sunlight absorption or had a gastrointestinal malabsorption disorder.

### Assessment

As part of the routine EDP assessment protocol, all children and adolescents had a comprehensive assessment consisting of a diagnostic evaluation with the patient and family by a trained pediatric psychiatrist or psychologist using the DSM-IV criteria (30), a complete medical assessment by an adolescent medicine specialist, and a nutritional assessment by a dietician with experience in pediatric eating disorders. Date of enrollment into the study was noted as the date of the assessment.

### Demographics, Historical, and Clinical Features

The full clinical assessment consisted of demographic information, a complete history of the eating disorder, past and current medication and supplement use including vitamin and calcium supplements, a full physical examination including Tanner staging and a detailed dietary history, as per the standard eating disorder assessment protocol at SickKids (30). A validated food frequency questionnaire was used to assess daily calcium and vitamin D intake (31).

### Anthropometrics

At the initial assessment, anthropometric measurements included weight (kg) and height (m) measured in a hospital gown, post void. All weights and heights were obtained on the same scale and stadiometer, respectively. BMI was calculated (kg/m^2^) as well as BMI percentiles.

### Laboratory Measurements

At time of enrollment, a blood and urine sample were obtained. All biological samples were assessed in the Department of Pediatric Laboratory Medicine (DPLM) laboratory at SickKids. Standard tests were used to determine serum albumin, calcium, phosphate, creatinine, urea, luteinizing hormone (18), follicle stimulating hormone (FSH), estradiol (E2), cortisol, thyroid stimulating hormone (TSH) and free thyroxine (fT4). Serum 25OHD was assessed with liquid chromatography-tandem mass spectrometry and intact PTH (iPTH) was assessed on the IDS-ISYS Multi-Discipline automated analyzer (22). The IDS-iSYS PTH assay is an immunoassay utilizing chemiluminescence technology wherein a polyclonal antibody captures the analyte at the C-terminus followed by an acridnium conjugated capture antibody targeted toward the N-terminus of the analyte (22). Both the full length, as well as the large PTH fragment (7-84) are detected. PTH results have been compared with age specific reference ranges from CALIPER (21). Calcium was determined by colorimetric assay using Arsenazo III dye on a chemistry analyzer and corrected to serum albumin. Vitamin D sufficiency was defined as a 25OHD level > 75 nmol/L, insufficiency as a serum 25OHD between 50 and 75 nmol/L and deficiency as <50 nmol/L. BMD (*n*= 34), LH (*n* = 42), FSH (*n* = 42), E2 (*n* = 36), morning cortisol (*n* = 22), TSH (*n* = 54), and fT4 (*n* = 54) were not measured as part of the study protocol. Lumbar spine (L1 to L4) areal BMD (aBMD) was measured by dual-energy X-ray absorptiometry (DXA) in the anterior-posterior direction (Lunar Prodigy; General Electric; Madison, WI, USA). The aBMD was transformed into Z-scores using the equipment specific age- and sex-adjusted Canadian reference values for aBMD (32).

### Statistical Analyses

Descriptive statistics were calculated for demographic, clinical and laboratory test variables. Mean plus standard deviation (SD) and range (minimum and maximum values) were provided for continuous variables. Count and proportions were calculated for categorical variables. Correlation analysis was performed to assess associations between PTH as a continuous variable and variables of interest including 25OHD, corrected calcium, total dietary calcium intake and urinary calcium/creatinine (Ca/Cr) ratio. In cases where non-linearity was observed, loess curves were used to visually estimate the approximate locations at which PTH changed magnitude of slope and/or direction. The effect sizes were expressed as sample correlation. The results were reported with 95% confidence interval (CI). A *p* < 0.05 was considered significant. All analyses were performed using SAS 9.4 software (SAS Institute Inc., Cary, N.C., USA).

### Statement of Ethics

The study was approved by the Research Ethics Board at SickKids. Standard institutional informed consent and assent procedures were followed in accordance with the Canadian Tri-Council Policy Statement.

## Results

A total of 61 patients (54 females, 7 males) with AN, with a mean age of 15.2 ± 1.56 years were included in the study. Table 1 outlines the relevant demographic and clinical characteristics at the initial assessment including calcium and vitamin D intake on admission, Tanner staging and lumbar spine BMD. Total calcium intake met or exceeded the recommended daily intake (RDI) of 1,200 mg per day in 56% of patients and was less than the RDI in 44% (33). Vitamin D intake was between 600 and 1,000 IU per day in 8%, <600 in 75% and <1,000 IU in 17%.

**Table 1:** Demographic Characteristics, BMD, and current Vitamin D and Calcium intake.

**Characteristic**	**Mean ± SD or *N (%)***	**Median**	**Range (min, max)**
Age	15.2 (1.56)		11.78–17.61
**Gender**			
Female	54 (89%)		
Male	7 (11%)		
**Diagnosis**			
ANR	49 (80%)		
ANBP	12 (20%)		
Weight (kg)	42.48 (6.01)	41.7	26.56–55.00
Height (m)	1.623 (0.076)	1.63	1.39–1.80
BMI (kg/m^2^)	15.97 (1.71)	15.9	11.90–18.80
BMI percentiles (%)	10.3 (12.7)	3.0	3.0–54.7
**Tanner staging (breast in female)**			
Tanner I	0 (0%)		
Tanner II	3 (5%)		
Tanner III	1 (2%)		
Tanner IV	2 (4%)		
Tanner II	3 (5%)		
Tanner V	48 (89%)		
Menarche	48 (89%)		
Secondary Amenorrhea ^+^	13 (24%)		
**Daily vitamin d intake (IU)^a^^,^^b^**			
Total	606.19 ± 771.69	359	0–4049.83
Dietary	135.01 ± 133.18	100	100–802
Supplements	471.18 ± 765.99	257	0–4000
Daily calcium intake (34)^a^^,^^b^	1047.38 ± 652.25	1052.86	0–2914
Spine BMD (L1-L4 z-score)	−0.69 (1.14)	−0.5	−2.70–1.40

ANR, Anorexia nervosa restrictive type; ANBP, Anorexia binge/purge type,

+ no period for 6 months or more. BMD, bone mineral density,

a For daily vitamin D and daily calcium intake, data were available from 59 of the 61 subjects.

b *Recommended daily intake for vitamin D 600–1,000 IU and for calcium 1,200 mg (33)*.

Mean E2 = 83.7 ± 58.4 pmol/l (*n* = 36), mean LH =1.7 ± 2.8 U/l and mean FSH = 3.4 ± 2.6 U/l were within age norms for randomly obtained samples (*n* = 42); mean TSH = 1.9 ± 1.5 U/l and mean fT4 = 11.0 ± 1.8 pmol/l were within the normal range (*n* = 54); and mean morning cortisol (454.4 ± 141.9 nmol/l) was high (*n* = 22).

Serum calcium, phosphate, 25OHD, PTH and related biochemistries are presented in Table 2. Thirty-five percent (*n* = 21) of the patients had PTH levels that were below the reference range for healthy boys and girls ages 9 to 17 years old (reference range 22–88 ng/L); differences were not observed between male and females and across Tanner stages. Overall, serum calcium (total and corrected) and 25OHD values were within the normal range and urinary Ca/Cr ratio was increased in our population.

**Table 2:** Patient Blood and Urine Biochemistry.

**Laboratory test**	**Mean ± SD or *N* (%)**	**Median**	**Range (min—max)**	**Reference range**
Total calcium (mmol/L)	2.39 ± 0.09	2.39	2.20–2.62	2.22–2.54
Corrected calcium (mmol/L)	2.30 ± 0.08	2.3	2.1–2.46	2.22–2.54
Albumin (g/L)	44.8 ± 3.8	45	37–53	35–53
Phosphate (mmol/L)	1.39 ± 0.19	1.4	0.70–1.80	1.00–1.63
Magnesium (mmol/L)	0.83 ± 0.06	0.82	0.70–1.01	0.70- 1.17
Creatinine (μmol/L)	63.72 ± 11.08	64.5	42–85	40–90
Urine Ca/Cr ratio (mmol/mmol)	0.87 ± 0.47	0.74	0.16–2.17	0.04-.7
25OHD (nmol/L)	98.81± 54.70	90	39–432	
<50	1 (2%)			Deficient
50–75	13 (25%)			Insufficient
>75	39 (74%)			sufficient
iPTH (ng/L)	28.42 ± 15.09	27	3–75	22–88^a^
<22	21 (35%)		3–21	low
22–88	39 (65%)		23–75	within range

All results had 53–61 samples analyzed. Lab reference ranges are approximate.

a *age specific reference range (21)*.

**Figure 1:**
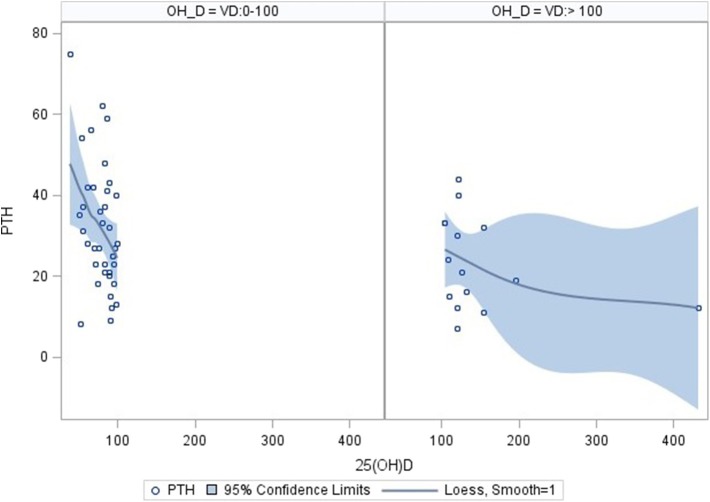
Correlation of PTH and 25OHD using inflection point of 100 nmol/l. Correlation analysis: PTH correlates significantly with 25OHD levels in the range 0–100 nmol/l (*n* = 38; parameter estimate −0.39, standard error 0.15, *p* = 0.0112). No significant correlation was found for 25OHD level above 100 nmol/l (*n* = 15; parameter estimate −0.04, standard error 0.04, *p* = 0.242). The pictures were produced with the PyMOL molecular graphics system (http://www.pymol.org).

Using correlation analysis, PTH had significant negative correlation with 25OHD below 100 nmol/l, but did not show a significant association with total or corrected serum calcium, urine Ca/Cr ratio, total dietary calcium intake, magnesium or Tanner staging (Table 3). The association between PTH and 25OHD was both negative and non-linear with an inflection point at a 25OHD level of 100 U/L, above which the association between the two values was no longer present (Figure 1).

**Table 3:** Correlation between PTH level and other variables.

	***n***	**Sample correlation**	**Lower 95% confidence limit**	**Upper 95% confidence limit**	***p*-value for H0:Rho = 0**
Serum calcium	60	0.00	−0.25	0.26	0.989
Corrected serum calcium	54	0.10	−0.18	−0.35	0.493
Urinary Ca/Crea ratio	54	−0.23	−0.46	0.05	0.102
Total calcium intake	58	−0.04	−0.29	0.22	0.778
Magnesium	60	0.24	−0.01	0.47	0.059
Tanner Staging^+^	53	−0.13	−0.39	0.14	0.345

+ *female subjects*.

## Discussion

### Main Finding

This is the first study to report low PTH levels in 35% of adolescents with AN using the CALIPER reference data for children between 9 and 17 years of age (21). These age-specific reference ranges are consistent with previous literature showing higher PTH levels in healthy children and adolescents compared to adults and preschool children (19, 20). No gender difference in PTH levels were noted in the adolescent age group from the CALIPER study (*n* = 534) as well as other studies (19, 21). Further and consistent with other studies in patients with AN, serum levels of calcium, phosphate and 25OHD were within the reference range and the urinary Ca/Cr ratio was increased (5, 12, 18).

### Interpretation and Comparison to Previous Literature

Consistent with our results, Haagensen et al. (18) showed lower PTH levels in girls (ages 15–26) with AN compared to healthy controls; the number of patients with PTH values below the reference range was not indicated in this study. Other studies reported no difference in PTH values when compared to controls (26, 28); however, investigators have used manufacturer reference ranges that cover the spectrum of values from childhood to adulthood, potentially masking a significant number of lower PTH levels (35).

The optimal range for PTH is one that maintains normal calcium homeostasis and bone turnover. There are several possible explanations for the reduced PTH levels that we observed in 35% of our patients with AN including: high calcium intake, magnesium deficiency, higher vitamin D levels and net loss of calcium from bone.

High dietary calcium intake has been described as a potential cause of low PTH levels (18). One study showed that 1 gram of oral elemental calcium resulted in a decrease of 25% or more in serum PTH levels among 10 of 12 healthy control subjects (aged 24–45 years) within 1–2 h of intake (36). In contrast, a study using calcium kinetic studies, showed reduced gastrointestinal calcium absorption in adolescent females with AN compared to healthy controls, suggesting that patients with AN may not exhibit a decrease in PTH levels in response to increased calcium intake (5). High exogenous calcium intake is likely not the reason for low PTH in our cohort given that dietary calcium was neither excessive nor did it correlate with PTH.

Magnesium deficiency has also been considered to be a potential cause of low PTH levels (37) as magnesium participates in PTH secretion and action through interaction with the calcium sensing receptor. Conditions of chronic magnesium depletion (as may be seen in severely malnourished patients with AN) can cause hypocalcemia with inappropriately normal or overtly low PTH levels. Magnesium and total serum calcium levels were normal in our cohort and no association was found in correlation analysis, rendering magnesium an unlikely explanation for the low PTH levels.

The importance of vitamin D and calcium in bone health is well-established and recommendations for the optimal intake of these nutrients is now widely accepted (7). Similar to previous reports in healthy individuals and in certain chronic diseases, the current study found a significant negative association between PTH and 25OHD in correlation analysis (38–42). The association was noted to be curvilinear with an inflection point at approximately 100 nmol/l of 25OHD visualized in loess curves. When the two component lines of this association were analyzed separately, only the line describing the association of PTH to 25OHD in the range of 0–100 nmol/l was significant. This finding is in keeping with previous literature and suggests that higher 25OHD levels do not contribute to the lower PTH levels (38).

Finally, a net loss of calcium from bone was found in calcium kinetic studies in adolescents with AN (5). The authors suggest that the demonstrated decrease rate in bone formation and increased rate in bone resorption is a possible explanation for the abnormalities in mineral metabolism observed in these patients (5). We did not perform calcium kinetic studies in our study population, however, the above mentioned mechanism is a possible explanation for the low PTH levels observed in our cohort.

### Limitations

One of the limitations of this study is the small sample size. This study was designed to look at central measures of calcium vitamin D metabolism in adolescents with newly diagnosed AN. However, the lack of data in the literature made it difficult to make assumptions about the sample size needed. As such, the results need to be interpreted carefully.

Several hormones (cortisol, LH and FSH) and DXA studies were measured as part of the clinical assessment and therefore were not consistently measured in all patients. These missing values limit detailed analysis of the associations between these factors and central measures of calcium and vitamin D metabolism and BMD. Nonetheless, where there were sufficient numbers, descriptive statistics have been calculated so as to provide the reader with the values found during the acute phase of AN.

### Potential Implications and Future Directions

The present study is the first to report low PTH levels in adolescents with AN when compared to values for PTH using an age-specific reference ranges (21). The present study lends support to the use of age-specific reference ranges in adolescents when evaluating PTH levels. The age-specific reference range may improve the accuracy of identifying low serum values of PTH in this age group. Our results suggest that low PTH levels may contribute to the pathogenesis of kidney stones as well as the bone phenotype in patients with AN by reducing bone formation and adding to the disturbance in mineralization.

This preliminary data are important for future research studies. Future studies with larger sample sizes are needed to corroborate our preliminary findings. Results from this study can be used to establish adequate sample size calculations in an effort to explore associations between PTH and hypercalciuria. Exploration of whether serum levels of vitamin D in the higher normal range and or 1,25OHD levels contribute to the suppression of PTH via subtle changes in ionized calcium or an alteration in the metabolism of vitamin D need to be investigated. Related to this, are studies assessing net calcium balance and bone mineralization in the patients with AN.

Lower PTH levels in adolescents with AN needs further investigation, given that this finding has potential clinical implications for both renal and bone health. Renal stones, nephrocalcinosis and renal failure have been reported in adults with AN (8, 43, 44). Adolescents with AN may be at risk of future renal complications given the reported reduced estimated glomerular filtration rate (9). Hypoparathyroidism is known to be associated with hypercalciuria, nephrocalcinosis, and kidney stones (45). In addition, our study shows that his population has increased urinary Ca/Cr ratio on a 24 h urine collection (5). Whether the reduced PTH levels observed in our population contribute to hypercalciuria and renal compromise needs further exploration.

The association between low bone mass and adolescents with AN is well-documented (7). Low IGF-1 levels due to GH resistance (46), low sex hormone levels due to functional hypogonadotropic hypogonadism (33), elevated cortisol (47) and low leptin levels (22) all contribute to low bone mass observed in patients with AN. However, the role of PTH levels in bone health in patients with AN remains less clear. Classical hypoparathyroidism in turn is characterized by a reduction in bone modeling, bone formation rate and mineral apposition (48, 49). These bone findings overlap significantly with what has been documented in patients with AN (5, 12, 13). As such, persistently low PTH levels may contribute to low bone formation and increased bone resorption in patients with AN. Further studies are needed to clarify the role of low PTH levels a bone health of patients with AN.

## Conclusion

This is the first study to report on low serum PTH levels in 35% of newly diagnosed adolescents with AN using the age-specific CALIPER reference data for children between 9 and 17 years of age (21). This finding is important in that it may provide a potential role for reduced PTH levels in the pathogenesis of kidney stones and bone phenotype in patients with AN.

## Data Availability Statement

The datasets generated for this study are available on request to the corresponding author.

## Ethics Statement

The studies involving human participants were reviewed and approved by Research Ethics Board at the Hospital for Sick Children. Written informed consent to participate in this study was provided by the participants' legal guardian/next of kin.

## Author Contributions

NL-T and KT have substantially contributed to this study including data curation, formal analysis, and drafting the initial manuscript. DK and ES have substantially contributed to this study including study design, methodology, investigations, supervision, funding acquisition, and reviewing and editing the manuscript.

### Conflict of Interest

The authors declare that the research was conducted in the absence of any commercial or financial relationships that could be construed as a potential conflict of interest.
